# Anomeric and Enantiomeric 2’‐Deoxycytidines: Base Pair Stability in the Absence and Presence of Silver Ions

**DOI:** 10.1002/chem.202101253

**Published:** 2021-06-10

**Authors:** Aigui Zhang, Simone Budow‐Busse, Peter Leonard, Frank Seela

**Affiliations:** ^1^ Laboratory of Bioorganic Chemistry and Chemical Biology Center for Nanotechnology Heisenbergstrasse 11 48149 Münster Germany; ^2^ Laboratorium für Organische und Bioorganische Chemie Institut für Chemie neuer Materialien Universität Osnabrück Barbarastrasse 7 49069 Osnabrück Germany

**Keywords:** anomers, deoxycytidine, enantiomers, DNA duplexes, oligonucleotides, silver

## Abstract

Dodecamer duplex DNA containing anomeric (α/β‐d) and enantiomeric (β‐l/β‐d) 2’‐deoxycytidine mismatches was studied with respect to base pair stability in the absence and presence of silver ions. Stable duplexes with silver‐mediated cytosine–cytosine pairs were formed by all anomeric and enantiomeric combinations. Stability changes were observed depending on the composition of the mismatches. Most strikingly, the new silver‐mediated base pair of anomeric α‐d‐dC with enantiomeric β‐l‐dC is superior to the well‐noted β‐d/β‐d‐dC pair in terms of stability. CD spectra were used to follow global helical changes of DNA structure.

## Graphical abstract

**Set‐in‐the‐silver C**: The base pair stability of anomeric (α/β‐d) and enantiomeric (β‐l/β‐d) 2’‐deoxycytidine mispair combinations in duplex DNA was studied in the absence and presence of silver ions. Stable duplexes with Ag^+^‐mediated cytosine–cytosine pairs were formed by all anomeric and enantiomeric combinations. The Ag^+^‐mediated base pair of anomeric α‐d‐dC (α‐d‐2’‐deoxycytidine) with enantiomeric β‐l‐dC (β‐l‐2’‐deoxycytidine) is superior to the well‐noted β‐d/β‐d‐dC pair in view of stability and *T*_m_ increase.

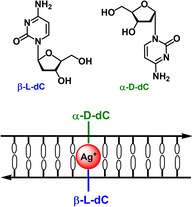

Enantiomeric l‐ and d‐DNA duplexes of identical sequence show identical physical properties in an achiral environment, including solubility, base pairing properties, thermal stability and hybridization.[^1^, ^2^] In contrast to d‐DNA, l‐DNA shows increased stability against DNA‐degrading enzymes.^[3]^ Consequently, bio‐orthogonal l‐DNA based technologies found novel applications in research and medicinal therapy.[^1^, ^4^] Awkwardly, oligonucleotides with β‐l‐configuration do not form stable heterochiral duplexes with oligonucleotides in the β‐d configuration.^[5]^ Nevertheless, single incorporations of l‐nucleosides in a Watson–Crick d‐DNA duplex are tolerated in such way that the overall B‐helix structure and the pairing selectivity is kept intact.[^3b^, ^6^] Also, nonamer duplexes containing silver‐mediated β‐l/β‐d‐dC mismatches in terminal or central positions have been reported^[7]^ (for structures of anomeric and enantiomeric cytidines; Figure 1). For anomeric DNA with one strand in β‐d and the other in α‐d configuration the situation is different. Anomeric oligonucleotides form stable duplexes with Watson–Crick type base pairs and a double helix with parallel strand orientation. Special protocols have been developed for the synthesis of α‐d nucleoside building blocks by stereospecific glycosylation protocols or anomerization of β‐d nucleosides. All four α‐d‐nucleosides as well as building blocks for DNA synthesis have been synthesized.^[8]^ Modified nucleosides have been incorporated in anomeric heterochiral DNA to stabilize the helix structure or to change the recognition pattern.^[9]^ In the presence of silver ions, silver‐mediated base pairing of α‐d‐dC with β‐d‐dC has been observed.^[10]^

**Figure 1:**
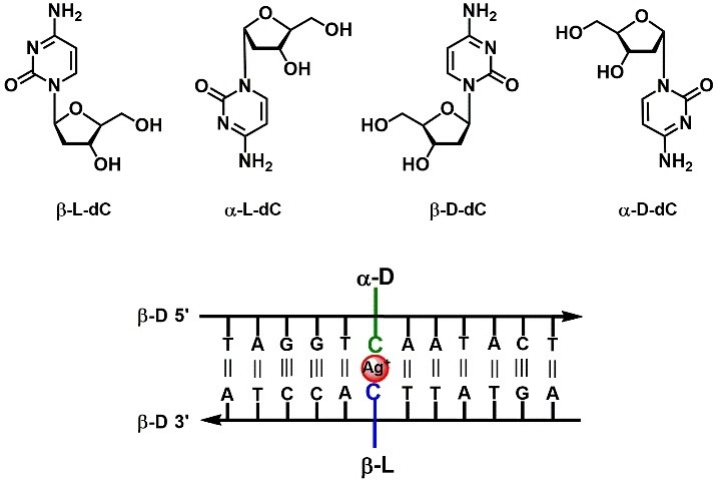
Top: Structures of anomeric (α/β) and enantiomeric (d/l) 2’‐deoxycytidine nucleosides. Bottom: Schematic representation of an antiparallel stranded duplex containing a silver‐mediated heterochiral β‐l/α‐d cytosine pair.

In this work, 12‐mer DNA duplexes with cytosine mispairs were constructed by combinations of anomeric and enantiomeric 2’‐deoxycytidines (Figure 1, bottom) and their stability was compared. Usually incorporation of noncanonical base pairs (mispairs) in homochiral or heterochiral DNA leads to a decrease of duplex stability. The decrease strongly depends on the particular structure of the mispair. Unpredictable interactions (hydrogen‐bonding, stacking) for each mispair have to be taken into account. This phenomenon is even stronger when silver ions are added and silver‐mediated base pairs are formed.

Earlier, the impact and stability of dC‐dC mispairs in duplex DNA has been reported.[^10^, ^11^] It was observed that the incorporation of the silver‐mediated anomeric β‐d/α‐d‐dC pair results in higher duplex stability than in case of the β‐d/β‐d‐dC pair.^[10]^ Therefore, we anticipated that the pair of anomeric α‐dC with enantiomeric β‐l‐dC might develop unexpected binding forces in the absence and the presence of silver ions. To this end, corresponding oligonucleotides were prepared. For the experimental details, including oligonucleotide synthesis and purity control, see the Supporting Information.

*T*_m_ values and thermodynamic data are commonly used to characterize the stability of DNA. These data are obtained from melting profiles according to thermal UV absorption changes using the two‐state model. Figure 2a and b display cooperative melting profiles of duplexes containing β‐l/α‐d‐dC (ODN‐**6⋅**ODN‐**7**) or β‐l/β‐d‐dC (ODN‐**6⋅**ODN‐**3**) base pairs in the presence and absence of silver ions. In Figure 2c, the stability of all β‐l‐dC base pair combinations is compared to the β‐d/β‐d‐dC pair in the presence of silver ions.

**Figure 2:**
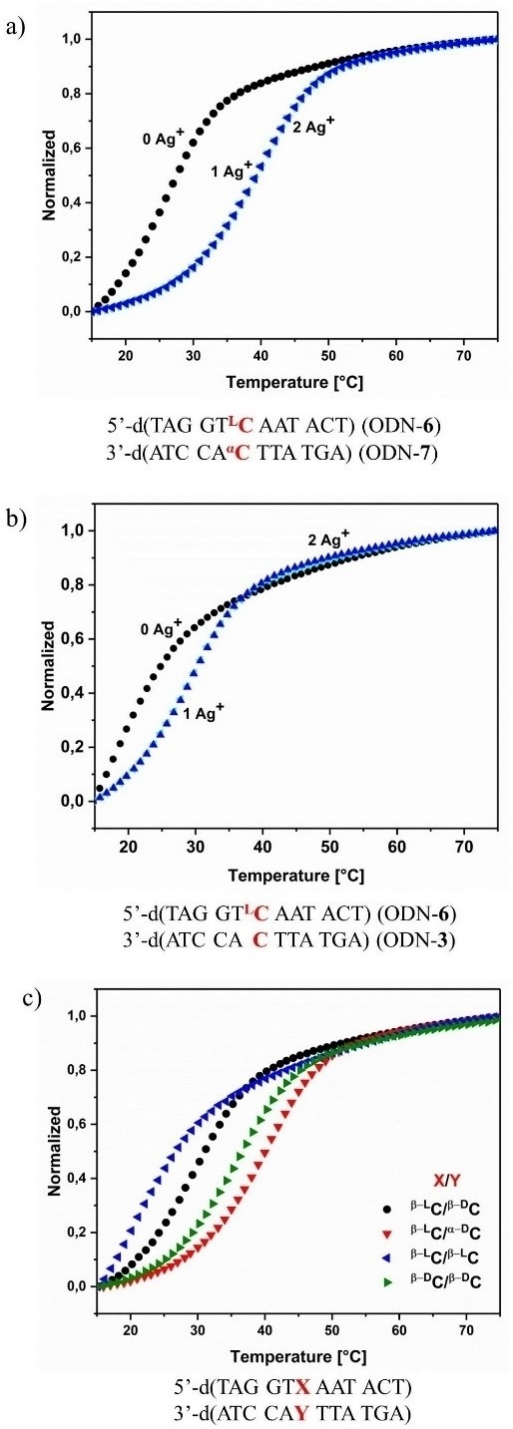
UV melting curves of oligonucleotide duplexes. a) ODN‐**6⋅**ODN‐**7** and b) ODN‐**6⋅**ODN‐**3** in the presence of various concentrations of Ag^+^ (0, 1, 2 silver ions/duplex). c) UV melting curves of oligonucleotide duplexes with different base combinations in the presence of 1 Ag^+^‐ion/duplex. Measured at 260 nm with 5 μM+5 μM single‐strand concentration at a heating rate of 1.0 °C/min in 100 mM NaOAc, 10 mM Mg(OAc)_2_, pH 7.4.

From Figure 2a it is obvious that in the absence of silver ions the duplex ODN‐**6⋅**ODN‐**7** containing a β‐l/α‐d‐dC pair is significantly more stable than duplex ODN‐**6⋅**ODN‐**3** comprising a β‐l/β‐d‐dC pair. Moreover, duplex stabilization upon silver ion addition is more pronounced for the β‐l/α‐d‐dC combination than for the β‐l/β‐d‐dC pair. The stability of duplex ODN‐**6⋅**ODN‐**7** is even higher than that of ODN‐**1⋅**ODN‐**3** incorporating the β‐d/β‐d‐dC pair (Figure 2c). Such a strong positive impact of the β‐l/α‐d‐dC pair was unexpected as the silver‐mediated β‐l/α‐d‐dC pair consists of a mispair formed by an unique combination of anomeric and enantiomeric dC residues. Apparently, the β‐l/α‐d‐dC pair is better accommodated in the DNA double helix compared to the β‐l/β‐d‐dC and β‐d/β‐d‐dC mispairs. Inspired by these results, the studies were extended to a series of other anomeric and enantiomeric 2’‐deoxycytidine base pair combinations. The results are set into relation to each other and *T*_m_ values are displayed in Table 1.

**Table 1:** *T*_m_ values of duplexes containing homochiral and heterochiral cytosine pairs in the absence or presence of silver ions.^[a]^

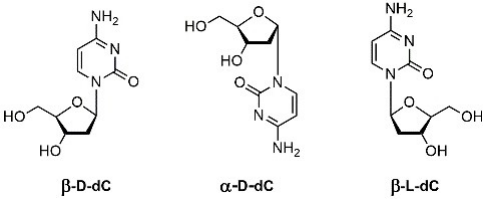		
Oligonucleotide duplex	*T*_m_ [°C]	*T*_m_ [°C]+1 Ag^+^/ds
		(Δ*T*_m_ [°C])
5’‐d(TAG GT **C** AAT ACT) (ODN‐**1**) 3’‐d(ATC CA **G** TTA TGA) (ODN‐**2**)	47.0	48.0 (+1)
5’‐d(TAG GT **C** AAT ACT) (ODN‐**1**) 3’‐d(ATC CA **C** TTA TGA) (ODN‐**3**)	26.5^[10a]^	34.0 (+7.5)
5’‐d(TAG GT^**α**^**C** AAT ACT) (ODN‐**4**) 3’‐d(ATC CA^**L**^**C** TTA TGA) (ODN‐**5**)	29.0	37.0 (+8.0)
5’‐d(TAG GT^**L**^**C** AAT ACT) (ODN‐**6**) 3’‐d(ATC CA^**α**^**C** TTA TGA) (ODN‐**7**)	28.0	39.0 (+11.0)
5’‐d(TAG GT **C** AAT ACT) (ODN‐**1**) 3’‐d(ATC CA^**α**^**C** TTA TGA) (ODN‐**7**)	29.5^[10a]^	43.5 (14.0)
5’‐d(TAG GT^**α**^**C** AAT ACT) (ODN‐**4**) 3’‐d(ATC CA **C** TTA TGA) (ODN‐**3**)	28.0^[10a]^	43.0 (15.0)
5’‐d(TAG GT **C** AAT ACT) (ODN‐**1**) 3’‐d(ATC CA^**L**^**C** TTA TGA) (ODN‐**5**)	<15^[b]^	29.0 (+>14.0)
5’‐d(TAG GT^**L**^**C** AAT ACT) (ODN‐**6**) 3’‐d(ATC CA **C** TTA TGA) (ODN‐**3**)	<15^[b]^	29.0 (+>14.0)
5’‐d(TAG GT^**L**^**C** AAT ACT) (ODN‐**6**) 3’‐d(ATC CA^**L**^**C** TTA TGA) (ODN‐**5**)	<15^[b]^	25.0 (+>10.0)
5’‐d(TAG GT^**α**^**C** AAT ACT) (ODN‐**4**) 3’‐d(ATC CA^**α**^**C** TTA TGA) (ODN‐**7**)	28.0^[10a]^	30.5 (+2.5)

[a] Measured at 260 nm with 5 μM+5 μM single‐strand concentration at a heating rate of 1.0 °C/min in 100 mM NaOAc, 10 mM Mg(OAc)2, pH 7.4 in the absence or presence of one silver ion/duplex. Tm values were calculated from the cooling curves using the program Meltwin 3.0.[12] Δ*T*_m_=*T*_m_ after the addition of AgNO_3_−*T*_m_ before the addition of AgNO3. [b] Exact Tm values could not be determined as no complete melting profiles were obtained in the range 15–50 °C. αC corresponds to α‐d‐2’‐deoxycytidine. LC corresponds to β‐l‐2’‐deoxycytidine.

According to the data obtained in the absence of silver ions (Table 1), the mispairs fall into two groups according to their stability (*T*_m_ values). Duplexes incorporating the base pair combinations β‐l/α‐d‐dC, β‐d/α‐d‐dC, β‐d/β‐d‐dC and α‐d/α‐d‐dC show *T*_m_ values around 26–29 °C. In contrast, the combinations β‐l/β‐d‐dC and β‐l/β‐l‐dC did not provide stable duplexes and single strands are present at ambient conditions (*T*_m_<15 °C). In the presence of silver ions, stable duplexes are formed and a significant *T*_m_ increase (Δ*T*_m_ 2.5–14 °C) is observed for all base pair combinations. Duplexes incorporating the new β‐l/α‐d‐dC base pair are stable even in the absence of silver ions and were strongly stabilized by silver ions *(T*_m_ increase 8–11 °C). The final *T*_m_ values of these duplexes are in the range of those with the β‐d/α‐d‐dC combination.

Beyond that, upon silver ion addition the β‐l/β‐d‐dC pair stabilized the duplex to the same extent (Δ*T*_m_=+14.0 °C) as observed for the β‐d/α‐d‐dC pair and stronger as for the well‐noted β‐d/β‐d‐dC combination.[^10^, ^11^] Significant duplex stabilization was also observed for the β‐l/β‐l‐dC combination (+10 °C), whereas for the α‐d/α‐d‐dC pair only a marginal *T*_m_ increase is apparent (+2.5 °C). Hence, configurational changes of the particular cytosine mismatches and binding forces to nearest‐neighbors (base pairing, stacking) have a strong impact on duplex stability.

To monitor the impact of the β‐l‐dC modification on the helical structure, CD measurements of the single strands and duplexes were performed (Figure 3a, Figures S11 and S12 in the Supporting Information). CD measurements constitute a valuable method to detect global helical changes in DNA structures when little material is available. For comparison, duplex spectra were calculated from the sum of the single strand spectra. The shape of the CD spectrum of single‐stranded ODN‐**6** (β‐l‐dC) is almost identical to that of the corresponding unmodified duplex ODN‐**1**. Only marginal differences in the amplitude are observed. The same is true for ODN‐**7** (α‐d‐dC) compared to ODN‐**2**. Upon duplex formation (with or without silver ions), the typical shape of a B‐DNA is observed. Helical distortion due to a particular mismatch would be also indicated by different CD curves obtained for experimental and calculated spectra. This is not the case (Figures 3 and S11). Thus, the anomeric/enantiomeric silver‐mediated dC/dC base pair combinations seem to fit nicely into the DNA double helix without changing its global structure.

**Figure 3:**
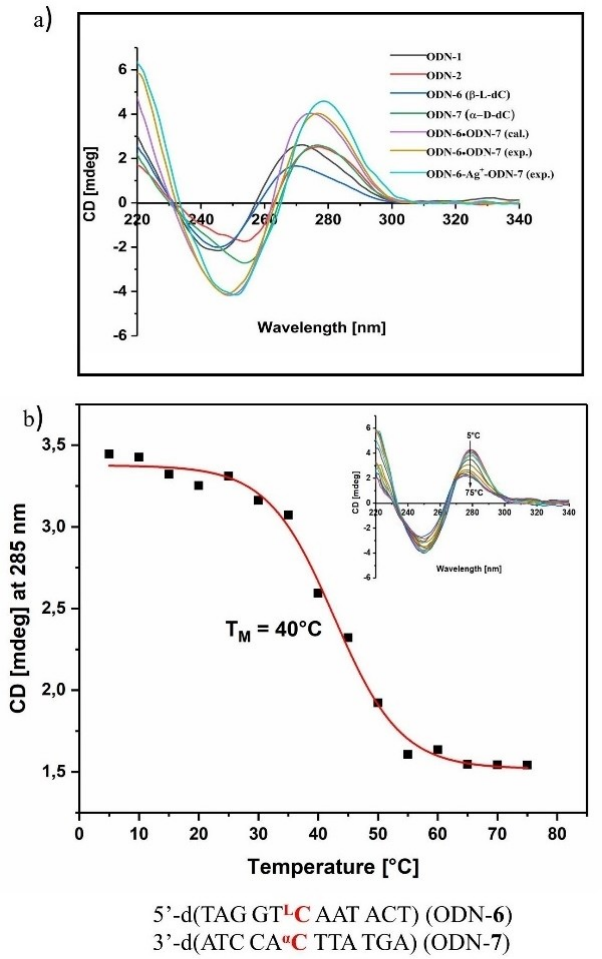
a) CD spectra of single strands ODN‐**6**, ODN‐**7**, duplex ODN‐**6⋅**ODN‐**7** (calculated and experimental) and ODN‐**6⋅**ODN‐**7** in the presence of 1 Ag^+^/duplex. b) CD melting curve of ODN‐**6⋅**ODN‐**7** in the presence of 1 Ag^+^/duplex obtained from temperature‐dependent CD spectra (inset). All measurements were performed in 100 mM NaOAc, 10 mM Mg(OAc)_2_ buffer (pH 7.4) with 5 μM+5 μM single‐strand concentration. The cell path length of the cuvette was 5 mm. ^**α**^ **C** corresponds to α‐d‐2’‐deoxycytidine. ^**L**^ **C** corresponds to β‐l‐2’‐deoxycytidine.

Next, temperature‐dependent CD spectra were measured, and from the spectral changes at a particular wavelength *T*_m_ values were determined (Figures 3b and S13). The wavelength for the CD melting profile has to be carefully chosen as the CD maxima are shifted upon duplex formation. For the duplex ODN‐**6⋅**ODN‐**7** containing the β‐l/α‐d‐dC pair, an almost identical *T*_m_ value (40 °C) to that obtained by UV melting (39 °C) was determined at 285 nm (Figure 3b). Also, for the other base pair combinations *T*_m_ values determined by CD spectra are in good agreement with those obtained by UV spectra (Figure S13).

The bar diagram of Figure 4 summarizes the *T*_m_ values of the various combinations of cytosine pairs in the presence and absence of silver ions. Most strikingly, it was found that the 2’‐deoxycytidine pair with one residue in α‐d and the other in β‐l configuration formed a silver‐mediated base pair almost as stable as the α‐d/β‐d‐dC combination. Both are superior to the well‐noted silver‐mediated pair in view of duplex stability and *T*_m_ increase. Remarkably, in the absence of silver ions possible duplexes with the mispair combinations β‐l/β‐d‐dC or β‐l/β‐l‐dC are not formed and only single strands are existing at ambient temperature. However, upon silver ion addition single strands were forced to duplex formation. For all other dC mispair combinations stable duplexes are already formed in the absence of silver ions and significant stability increases are observed in the presence of metal ions. Thus, we anticipate that lower‐melting duplexes form only silver bridges and not hydrogen bonds, whereas duplexes with higher *T*_m_ values form silver bridges and in addition hydrogen bonds. Base pairs with different base alignments are possible as discussed previously.^[10]^

**Figure 4:**
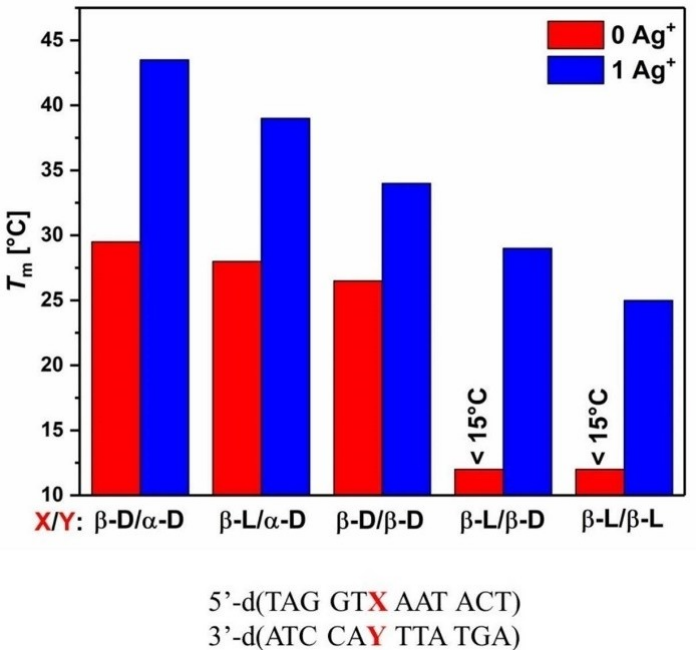
Bar diagram showing the *T*_m_ values of DNA duplexes containing cytosine pairs. Thermal UV melting was performed in the absence (red) and presence (blue) of one silver ion per duplex, according to Table 1.

The impact of anomeric and enantiomeric 2’‐deoxycytidine modifications on the global structure of single strands and duplexes was demonstrated by CD measurements. Whereas the CD maxima of the particular single strands with anomeric or enantiomeric dC residues are shifted, the typical shape of a B‐DNA was maintained upon duplex formation. Temperature‐dependent CD spectra of duplexes confirmed the *T*_m_ values obtained from UV melting profiles.

## Conflict of interest

The authors declare no conflict of interest.

## Supporting information

As a service to our authors and readers, this journal provides supporting information supplied by the authors. Such materials are peer reviewed and may be re‐organized for online delivery, but are not copy‐edited or typeset. Technical support issues arising from supporting information (other than missing files) should be addressed to the authors.

Supporting Information
