# Supplementary material for: Anomeric and Enantiomeric 2’‐Deoxycytidines: Base Pair Stability in the Absence and Presence of Silver Ions
Source: Chemistry. 2021 Jun 10;27(41):10574–7. doi: 10.1002/chem.202101253 (PMC8362019; doi:10.1002/chem.202101253)
Supplement: Supplementary file 1 — Supporting Information [file CHEM-27-10574-s001.pdf]

Chemistry–A European Journal

Supporting Information

Anomeric and Enantiomeric 2′-Deoxycytidines: Base Pair Stability in the Absence and Presence of Silver Ions

Aigui Zhang, Simone Budow-Busse, Peter Leonard, and Frank Seela*

Table of Contents

General methods and materials 2

Oligonucleotide syntheses and characterization 2

Figure S1. Structures of the phosphoramidites 3

Table S1. Synthesized oligonucleotides and their molecular masses determined by MALDI-TOF mass spectrometry 4

References 4

Figures S2-8. Reversed-phase HPLC profiles of oligonucleotides 5-7

Figure S9. Melting profiles of oligonucleotide duplexes in the absence of silver ions 7-9

Figure S10. Melting profiles of oligonucleotide duplexes in the absence and presence of silver ions 9-12

Table S2. T_m values of duplexes containing homochiral or heterochiral cytosine mispairs in the absence or presence of 0-2 silver ions 13

Figure S11. CD spectra of single strands, calculated duplex and experimental duplex 14-15

Figure S12. CD spectra of duplexes determined in the absence and presence of various concentrations of Ag^+ 15-17

Figure S13. Temperature-dependent CD spectra 18-20

Experimental

General methods and materials

All chemicals and solvents were of laboratory grade as obtained from commercial suppliers and were used without further purification. Reversed-phase HPLC was carried out on a 4 × 250 mm RP-18 (10 μm) LiChrospher 100 column with a HPLC pump connected with a variable wavelength UV monitor, a controller and an integrator. The molecular masses of the oligonucleotides were determined by MALDI-TOF mass spectrometry on a Bruker Autoflex Speed instrument in the linear positive mode with 3-hydroxypicolinic acid (3-HPA) as a matrix. Thermal melting curves were measured with an Agilent Technologies Cary 100 Bio UV/Vis spectrophotometer equipped with a thermoelectric controller. The temperature was measured continuously in the reference cell with a Pt-100 resistor, employing a heating rate of 1°C min^{-1}. T_m values were determined from the melting curves by using the software *Meltwin*, version 3.0.[1] CD-spectra were recorded at 25 °C on a JASCO J-815 spectrometer.

Oligonucleotide syntheses and characterization

Solid-phase oligonucleotide syntheses were performed on an ABI 392-08 synthesizer at 1 μmol scale (trityl-on mode) employing the phosphoramidite of β-L-dC and α-D-dC (for structures, see Figure S1) as well as the standard building blocks with an average coupling yield over 95%. The phosphoramidite of β-L-dC[2] was purchased from Chemgenes. The phosphoramidite of α-D-dC was prepared according to a literature protocol.[3] β-L-dC and α-D-dC were incorporated in the antiparallel duplex 5'-d(TAGGT**C**AATACT) (ODN-**1**) • 3'-d(ATCCA**G**TTATGA) (ODN-**2**), replacing a central dC-dG base pair. After cleavage from the solid support, oligonucleotides were deprotected in 28% aqueous ammonia at 55 °C for 2

h. The DMT-containing oligonucleotides were purified by reversed-phase HPLC (RP-18) with the gradient system at 260 nm: (A) MeCN, (B) 0.1 M (Et_3NH)OAc (pH 7.0)/MeCN, 95:5; gradient I: 0-3 min 10-15% A in B, 3-15 min 15-50% A in B; flow rate 0.8 mL/min. The purified “trityl-on” oligonucleotides were treated with 2.5% $CHCl_2COOH/CH_2Cl_2$ for 2 min at 0 °C to remove the 4,4′-dimethoxytrityl residues. The detritylated oligomers were purified again by reversed-phase HPLC with gradient II: 0-20 min 0-20% A in B; 20-25 min, 20% A 3in B; flow rate 0.8 mL/min. The oligonucleotides were desalted on short column (RP-18) using water for elution of salt, while the oligonucleotides were eluted with H_2O/MeOH (2:3). The oligonucleotides were lyophilized on a Speed-Vac evaporator to yield colourless solids which were frozen at -24 °C. The purity of all oligonucleotides was confirmed by RP-18 HPLC (Figure S2) and MALDI-TOF mass spectrometry (Table S1). The extinction coefficients ε_{260} (H_2O) of the nucleosides are: dA 15400, dG 11700, dT 8800, dC 7300, α-dC 7300, L-dC 7300. The extinction coefficients of the oligonucleotides were calculated from the sum of the extinction coefficients of nucleoside constituents.

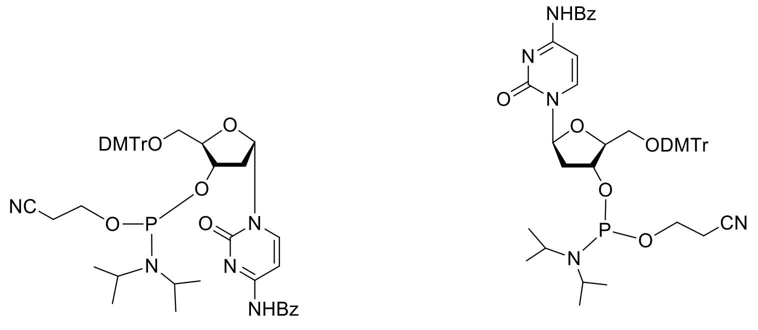

Phosphoramidite of α-D-dC **Phosphoramidite of β-L-dC**

Figure S1. Structures of the phosphoramidites of α-D-dC and β-L-dC.

Table S1. Synthesized oligonucleotides and their molecular masses determined by MALDI-TOF mass spectrometry.

Entry	Oligonucleotides	*Mr* calcd.[a] *Mr* found[b]	Entry	Oligonucleotides	*Mr* calcd.[a] *Mr* found[b]
ODN-**1**	5’-d(TAGGTCAATACT)[4]	3643.6 3643.7	ODN-**5**	5’-d(AGTATT$^{\alpha}$**C**ACCTA)[6]	3605.4 3603.3
ODN-**2**	5’-d(AGTATTGACCTA)[4]	3643.6 3643.7	ODN-**6**	5’-d(AGTATT^{L}**C**ACCTA)	3604.4 3604.4
ODN-**3**	5’-d(AGTATT**C**ACCTA)[5]	3603.6 3603.9	ODN-**7**	5’-d(TAGGT^{L}**C**AATACT)	3644.4 3643.7
ODN-**4**	5’-d(TAGGT$^{\alpha}$**C**AATACT)[3]	3645.4 3644.7			

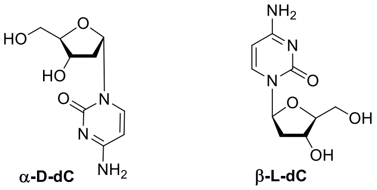

α-D-dC **β-L-dC**

[a] Calculated on the basis of the molecular mass of [*M* + H]$^+$. [b] Determined by MALDI-TOF mass-spectrometry as [*M* + H]$^+$ in the linear positive mode. $^{\alpha}$**C** corresponds to α-D-2’-deoxycytidine. ^{L}**C** corresponds to β-L-2’-deoxycytidine.

References

[1] J. A. McDowell, D. H. Turner, *Biochemistry* **1996**, *35*, 14077-14089.

[2] H. Urata, E. Ogura, K. Shinohara, Y. Ueda, M. Akagi, *Nucleic Acids Res.* **1992**, *20*, 3325-3332.

[3] Y. Chai, P. Leonard, X. Guo, F. Seela, *Chem. Eur. J.* **2019**, *25*, 16639-16651.

[4] H. Zhao, P. Leonard, X. Guo, H. Yang, F. Seela, *Chem. Eur. J.* **2017**, *23*, 5529-5540.

[5] X. Guo, F. Seela, *Chem. Eur. J.* **2017**, *23*, 11776-11779.

[6] Y. Chai, X. Guo, P. Leonard, F. Seela, *Chem. Eur. J.* **2020**, *26*, 13973-13989.

Reversed-phase (RP-18) HPLC profiles of oligonucleotides

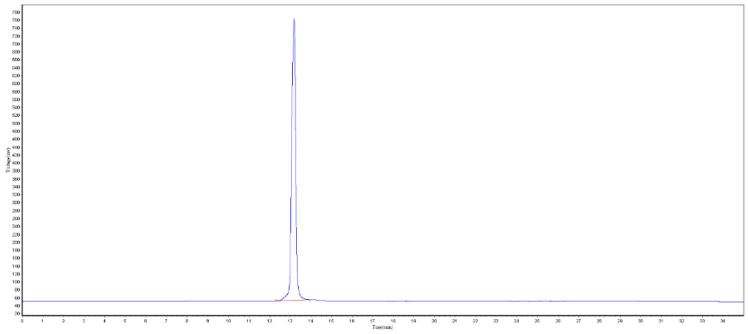

Figure S2. 5’-d(TAG GTC AAT ACT) (ODN-**1**)

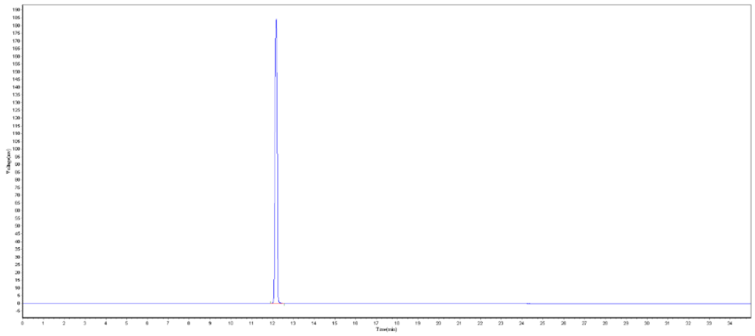

Figure S3. 5’-d(AGT ATT GAC CTA) (ODN-**2**)

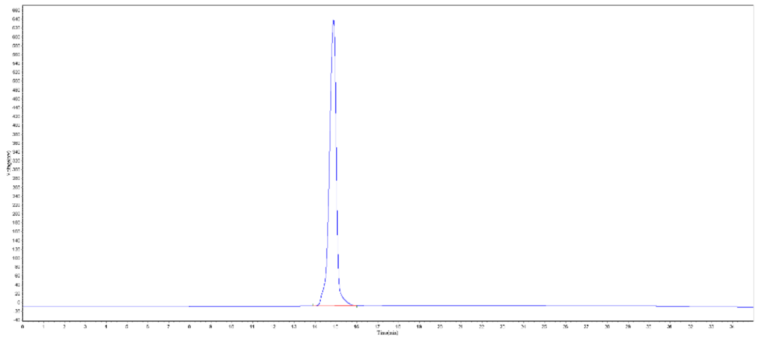

Figure S4. 5’-d(AGTATT**C**ACCTA) (ODN-**3**)

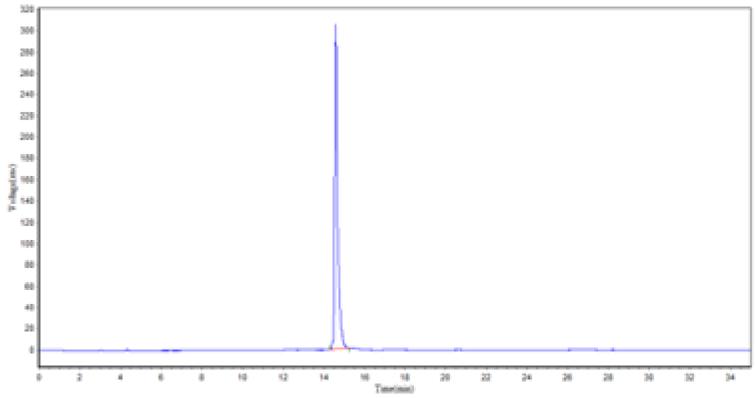

Figure S5. 5’-d(TAGGT$^{\alpha}$**C**AATACT) (ODN-**4**)

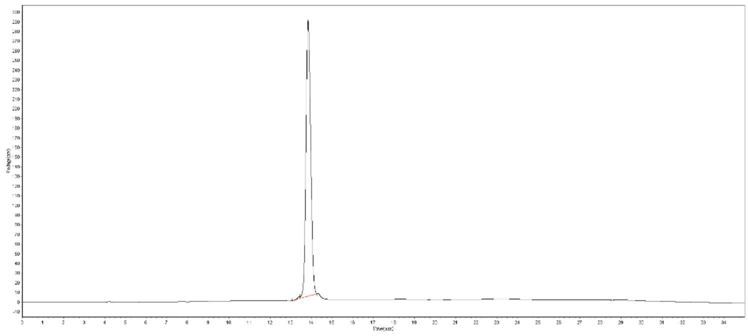

Figure S6. 5’-d(AGTATT^{L}**C**ACCTA) (ODN-**5**)

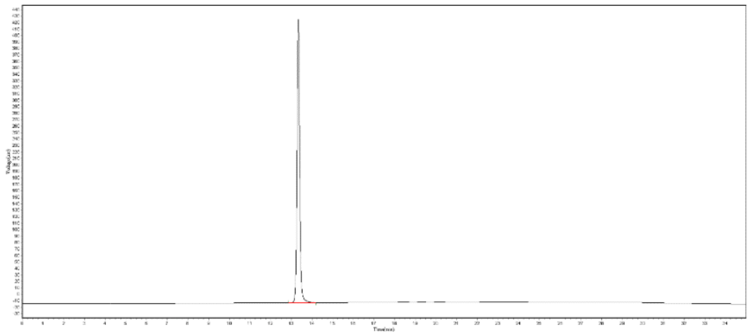

Figure S7. 5’-d(TAGGT^{L}**C**AATACT) (ODN-**6**)

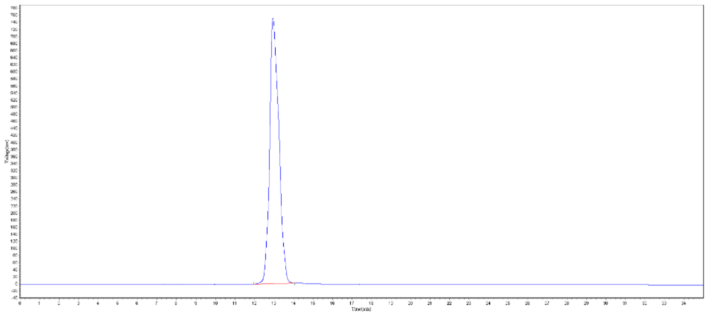

Figure S8. 5’-d(AGTATT$^{\alpha}$**C**ACCTA) (ODN-**7**)

Figures S2-8. Reversed-phase (RP-18) HPLC elution profiles of purified oligonucleotides monitored at 260 nm. X-axis refers to retention time (min); Y-axis refers to UV absorbance at 260 nm, measured in mV. For elution, the following system was used: (A) MeCN, (B) 0.1 M $(Et_3NH)OAc$ (pH 7.0)/MeCN, 95:5; gradient: 0-20 min 0-20% A in B; 20-25 min, 20% A in B; flow rate 0.7 mL/min.

Melting profiles of oligonucleotide duplexes in the absence of silver ions

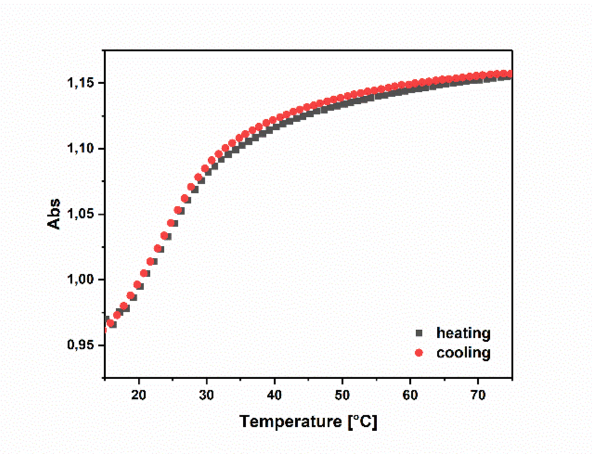

5’-d(TAG GT**C** AAT ACT) (ODN-**1**)
3’-d(ATC CA**C** TTA TGA) (ODN-**3**)

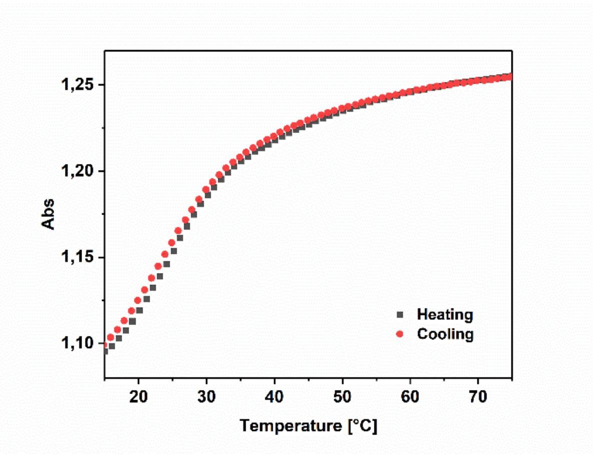

5’-d(TAG GT$^{\alpha}$**C** AAT ACT) (ODN-**4**)
3’-d(ATC CA^{L}**C** TTA TGA) (ODN-**5**)

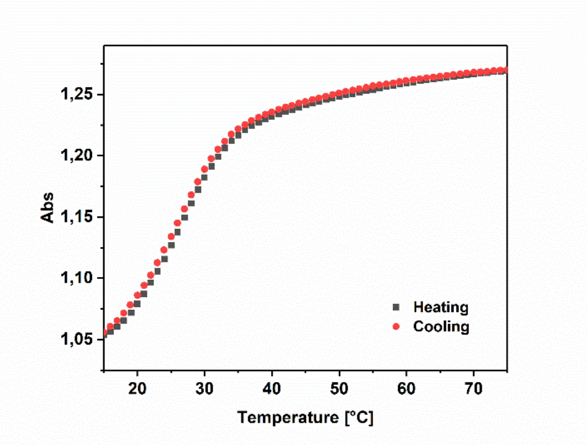

5’-d(TAG GT^{L}**C** AAT ACT) (ODN-**6**)
3’-d(ATC CA$^{\alpha}$**C** TTA TGA) (ODN-**7**)

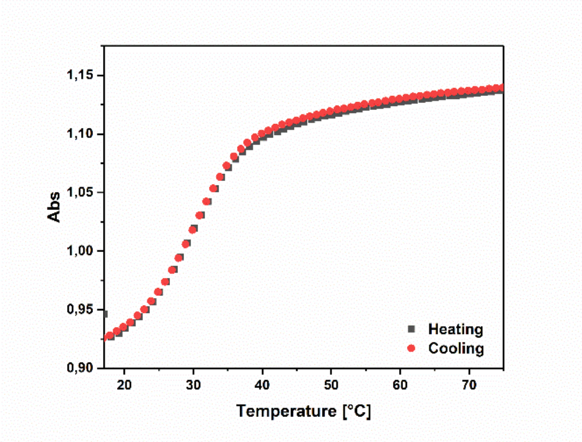

5’-d(TAG GT **C** AAT ACT) (ODN-**1**)
3’-d(ATC CA$^{\alpha}$**C** TTA TGA) (ODN-**7**)

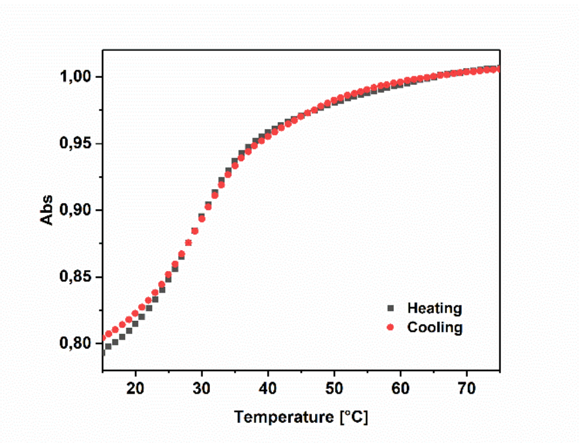

5’-d(TAG GT$^{\alpha}$**C** AAT ACT) (ODN-**4**)
3’-d(ATC CA **C** TTA TGA) (ODN-**3**)

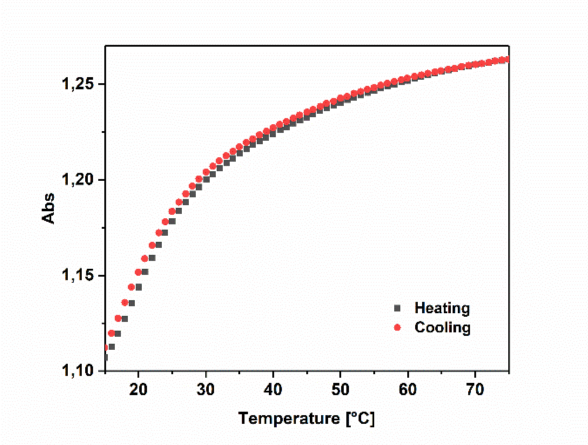

5’-d(TAG GT **C** AAT ACT) (ODN-**1**)
3’-d(ATC CA^{L}**C** TTA TGA) (ODN-**5**)

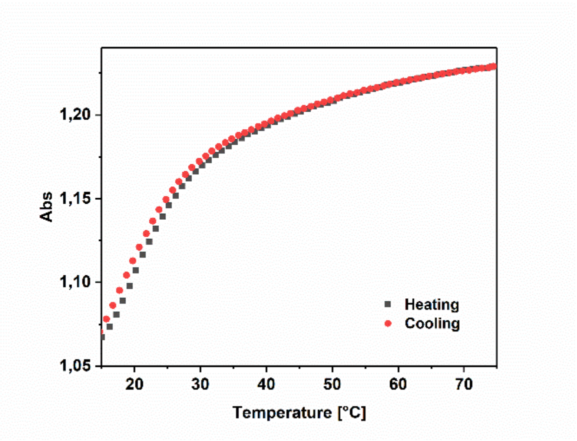

5’-d(TAG GT^{L}**C** AAT ACT) (ODN-**6**)
3’-d(ATC CA **C** TTA TGA) (ODN-**3**)

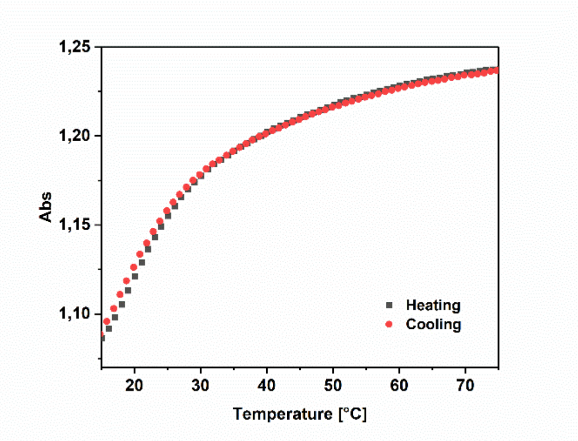

5’-d(TAG GT^{L}**C** AAT ACT) (ODN-**6**)
3’-d(ATC CA^{L}**C** TTA TGA) (ODN-**5**)

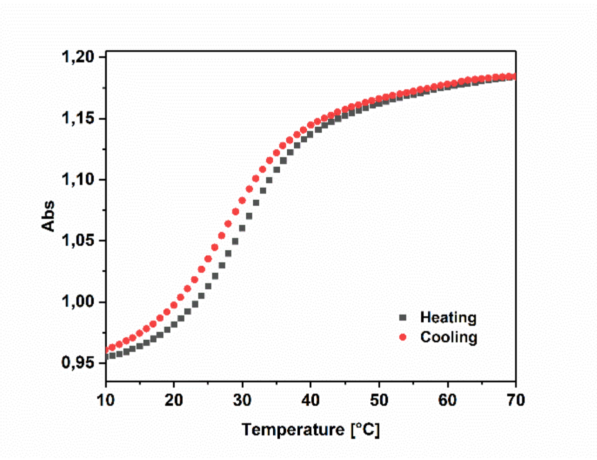

5’-d(TAG GT$^{\alpha}$**C** AAT ACT) (ODN-**4**)
3’-d(ATC CA$^{\alpha}$**C** TTA TGA) (ODN-**7**)

Figure S9. Thermal denaturation curves of duplexes measured at 260 nm with 5 µM + 5 µM single-strand concentration in 100 mM NaOAc, 10 mM $Mg(OAc)_2$ buffer (pH = 7.4) and a heating rate of 1.0°C/min.

Melting profiles of oligonucleotide duplexes in the absence and presence of silver ions

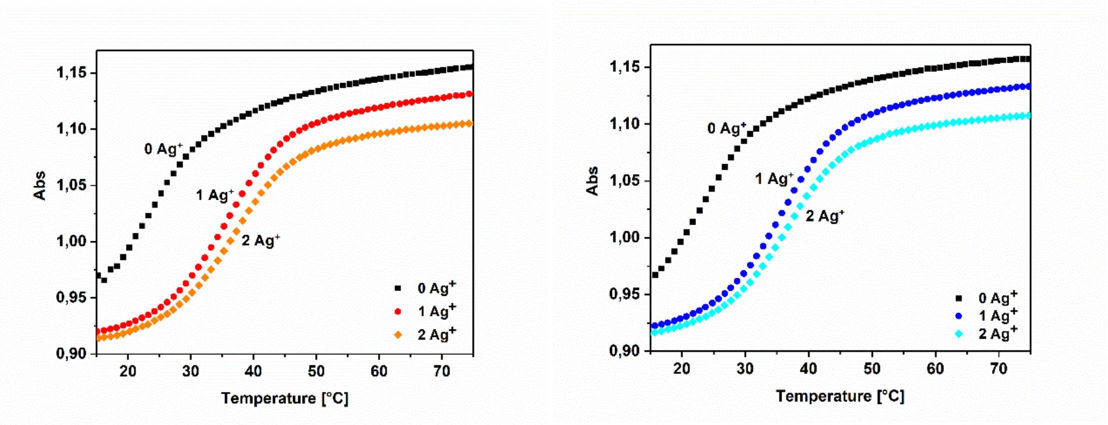

5’-d(TAG GT**C** AAT ACT) (ODN-**1**)
3’-d(ATC CA**C** TTA TGA) (ODN-**3**)

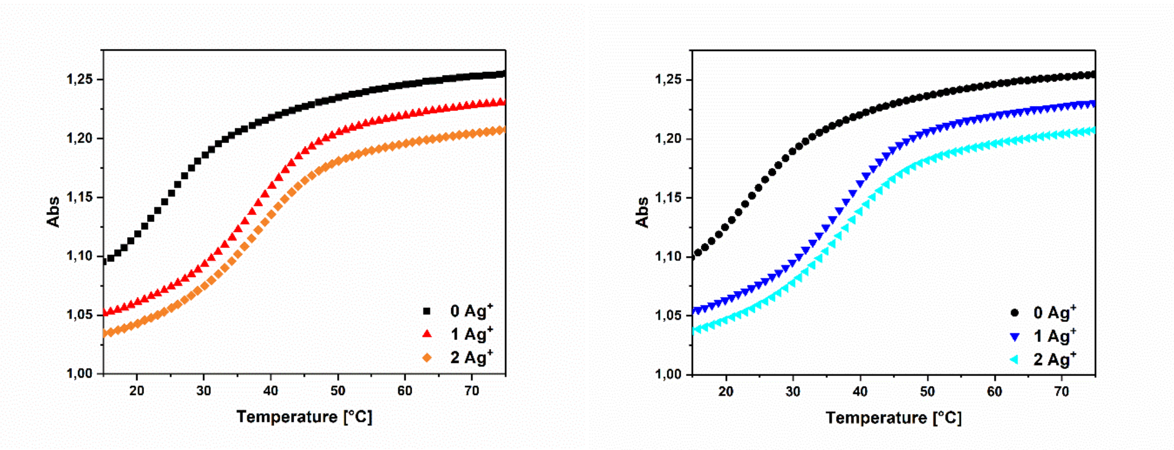

5’-d(TAG GT$^{\alpha}$**C** AAT ACT) (ODN-**4**)
3’-d(ATC CA^{L}**C** TTA TGA) (ODN-**5**)

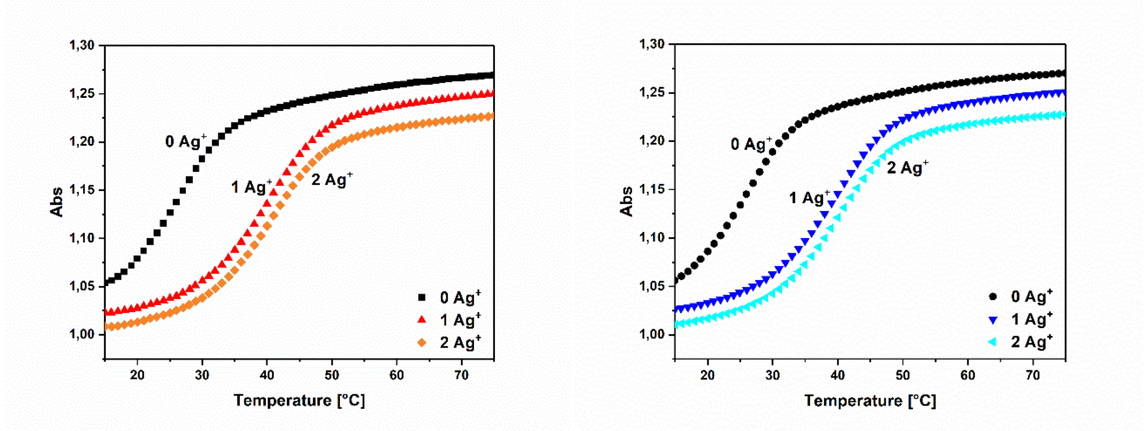

5’-d(TAG GT^{L}**C** AAT ACT) (ODN-**6**)
3’-d(ATC CA$^{\alpha}$**C** TTA TGA) (ODN-**7**)

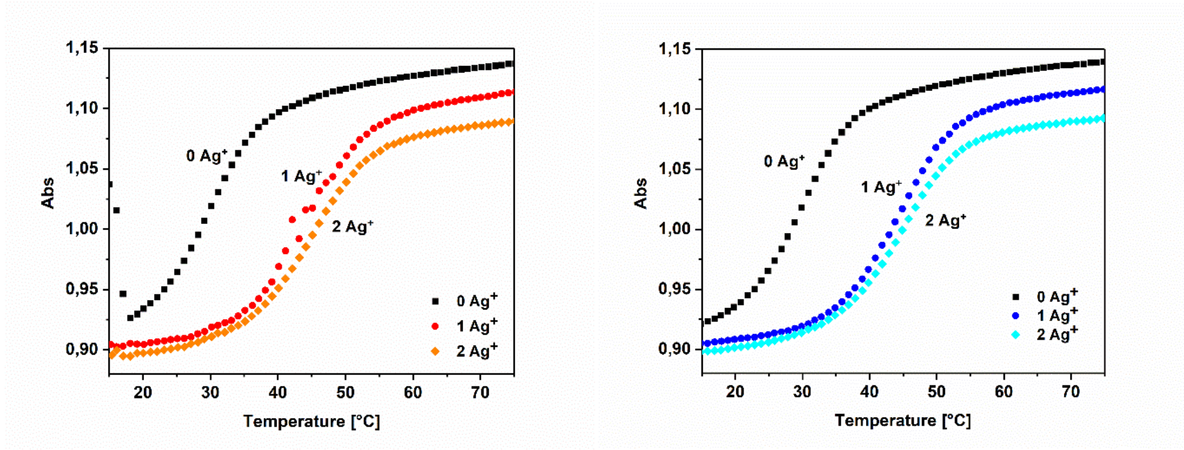

5’-d(TAG GT **C** AAT ACT) (ODN-**1**)
3’-d(ATC CA$^{\alpha}$**C** TTA TGA) (ODN-**7**)

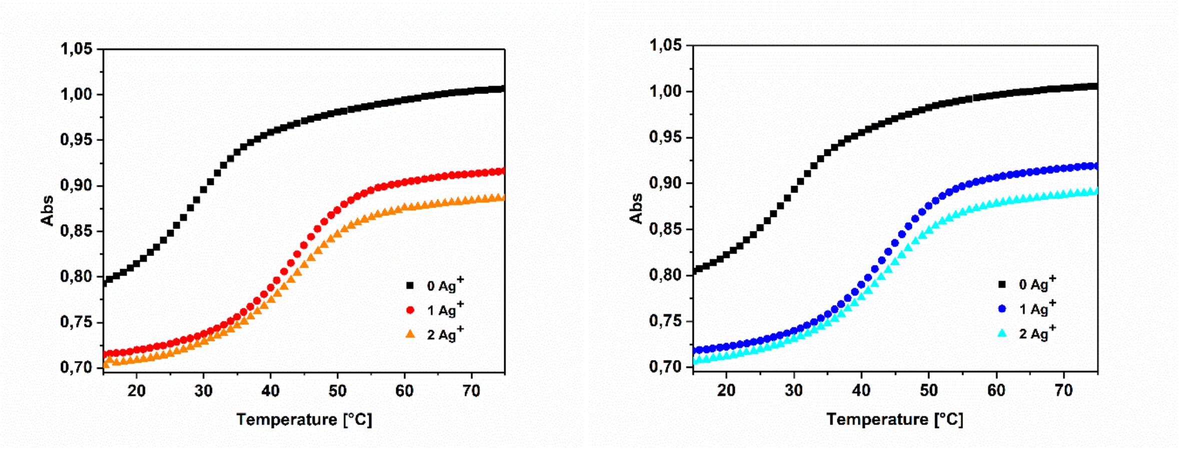

5’-d(TAG GT$^{\alpha}$C AAT ACT) (ODN-**4**)
3’-d(ATC CA C TTA TGA) (ODN-**3**)

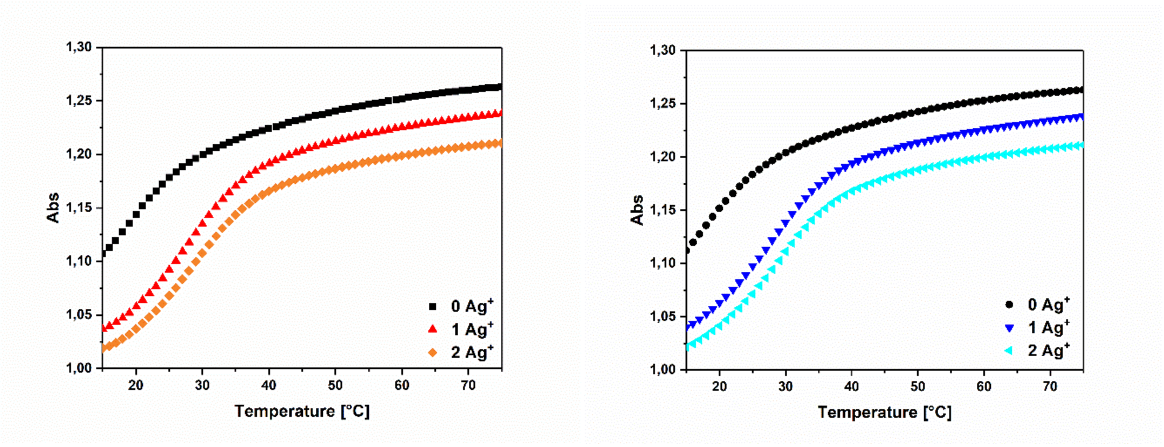

5’-d(TAG GT C AAT ACT) (ODN-**1**)
3’-d(ATC CA^{L}C TTA TGA) (ODN-**5**)

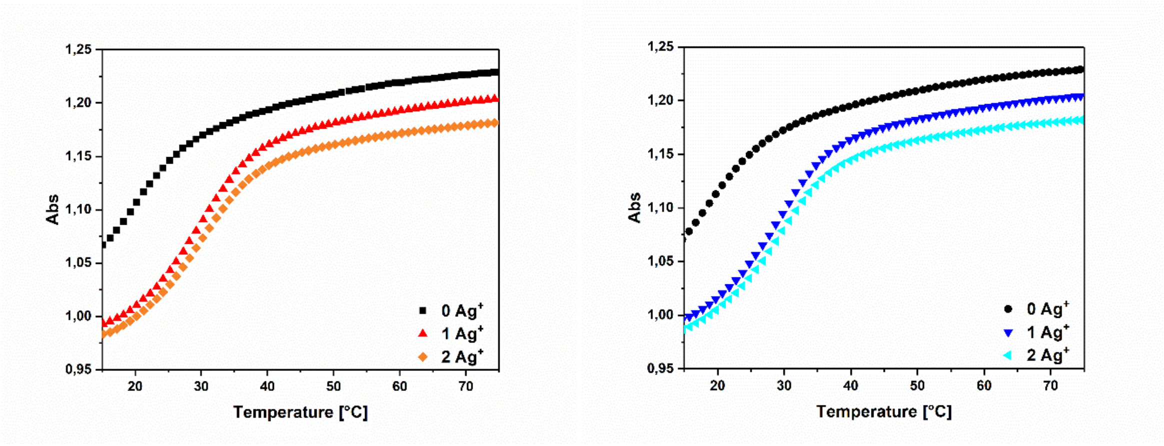

5’-d(TAG GT^{L}C AAT ACT) (ODN-**6**)
3’-d(ATC CA C TTA TGA) (ODN-**3**)

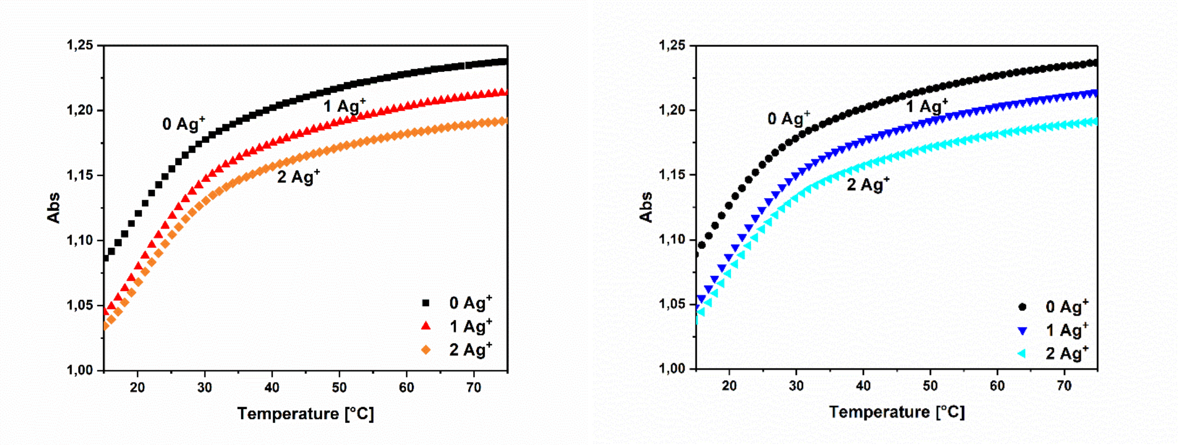

5’-d(TAG GT^{L}**C** AAT ACT) (ODN-**6**)
3’-d(ATC CA^{L}**C** TTA TGA) (ODN-**5**)

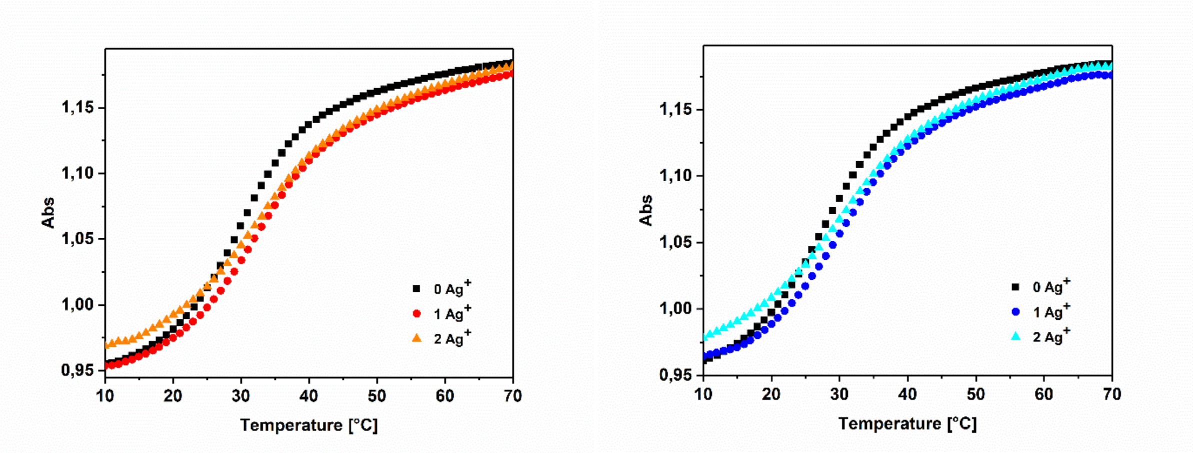

5’-d(TAG GT$^{\alpha}$**C** AAT ACT) (ODN-**4**)
3’-d(ATC CA$^{\alpha}$**C** TTA TGA) (ODN-**7**)

Figure S10. Thermal denaturation curves of duplexes measured at 260 nm with 5 µM + 5 µM single-strand concentration in 100 mM NaOAc, 10 mM $Mg(OAc)_2$ buffer (pH = 7.4) in the presence of various concentrations of Ag^+ (0, 1 and 2 silver ions/duplex) and a heating rate of 1°C/min. Left) Heating data. Right) Cooling data.

Table S2. T_m values of duplexes containing homochiral or heterochiral cytosine mispairs in the absence or presence of 0-2 silver ions.[a]

**Duplexes**	T_m [°C]	T_m [°C] +1 Ag^+/ds (ΔT_m [°C])	T_m [°C] +2 Ag^+/ds (ΔT_m [°C])
5'-d(TAG GT**C** AAT ACT) (ODN-**1**)<br>3'-d(ATC CA**G** TTA TGA) (ODN-**2**)	47.0	48.0<br>(+1)	n.m.
5'-d(TAG GT**C** AAT ACT) (ODN-**1**)<br>3'-d(ATC CA**C** TTA TGA) (ODN-**3**)	26.5[3]	34.0 (+7.5)	35.0 (+8.5)
5'-d(TAG GT$^{\alpha}$**C** AAT ACT) (ODN-**4**)<br>3'-d(ATC CA^{L}**C** TTA TGA) (ODN-**5**)	29.0	37.0 (+8.0)	37.0 (+8.0)
5'-d(TAG GT^{L}**C** AAT ACT) (ODN-**6**)<br>3'-d(ATC CA$^{\alpha}$**C** TTA TGA) (ODN-**7**)	28.0	39.0 (+11.0)	39.0 (+11.0)
5'-d(TAG GT **C** AAT ACT) (ODN-**1**)<br>3'-d(ATC CA$^{\alpha}$**C** TTA TGA) (ODN-**7**)	29.5[3]	43.5 (+14.0)	44.0 (+14.5)
5'-d(TAG GT$^{\alpha}$**C** AAT ACT) (ODN-**4**)<br>3'-d(ATC CA **C** TTA TGA) (ODN-**3**)	28.0[3]	43.0 (+15.0)	43.5 (+15.5)
5'-d(TAG GT **C** AAT ACT) (ODN-**1**)<br>3'-d(ATC CA^{L}**C** TTA TGA) (ODN-**5**)	<15[b]	29.0 (+ >14.0)	29.0 (+ >14.0)
5'-d(TAG GT^{L}**C** AAT ACT) (ODN-**6**)<br>3'-d(ATC CA **C** TTA TGA) (ODN-**3**)	<15[b]	29.0 (+ >14.0)	30.0 (+ >15.0)
5'-d(TAG GT^{L}**C** AAT ACT) (ODN-**6**)<br>3'-d(ATC CA^{L}**C** TTA TGA) (ODN-**5**)	<15[b]	25.0 (+ >10.0)	25.0 (+ >10.0)
5'-d(TAG GT$^{\alpha}$**C** AAT ACT) (ODN-**4**)<br>3'-d(ATC CA$^{\alpha}$**C** TTA TGA) (ODN-**7**)	28.0[3]	30.5 (+2.5)	30.5 (+2.5)

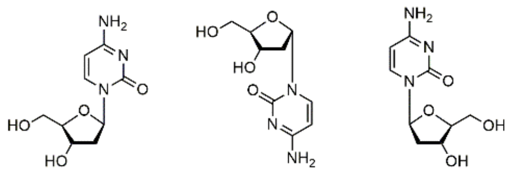

β-D-dC   α-D-dC   β-L-dC

[a] Measured at 260 nm with 5 µM + 5 µM single-strand concentration at a heating rate of 1.0 °C/min in 100 mM NaOAc, 10 mM $Mg(OAc)_2$, pH 7.4 in the absence or presence of one silver ion/duplex. T_m values were calculated from the cooling curves using the program *Meltwin 3.0*.[1] ΔT_m = T_m after the addition of $AgNO_3$ - T_m before the addition of $AgNO_3$. [b] Exact T_m values could not be determined as no complete melting profiles were obtained in the range 15-50°C. $^{\alpha}$**C** corresponds to α-D-2'-deoxycytidine. ^{L}**C** corresponds to β-L-2'-deoxycytidine. n.m. corresponds to not measured.

CD measurements

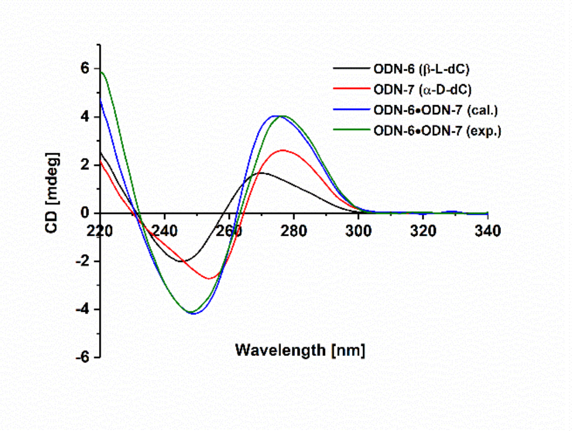

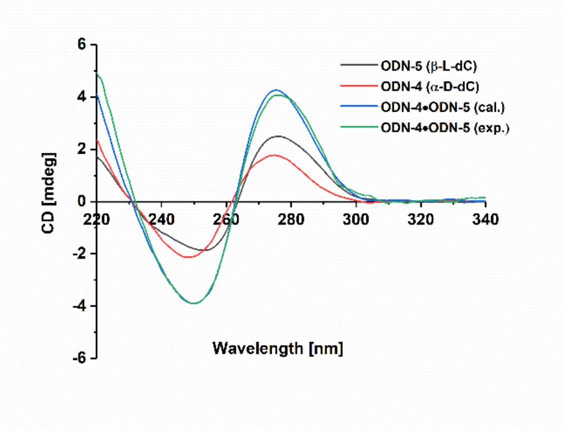

5’-d(TAG GT^{L}**C** AAT ACT) (ODN-**6**)
3’-d(ATC CA$^{\alpha}$**C** TTA TGA) (ODN-**7**)

5’-d(TAG GT$^{\alpha}$**C** AAT ACT) (ODN-**4**)
3’-d(ATC CA^{L}**C** TTA TGA) (ODN-**5**)

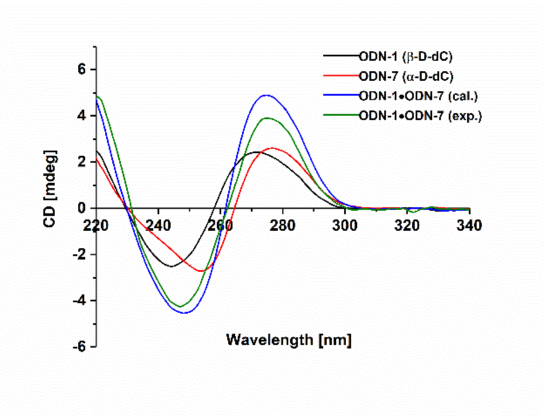

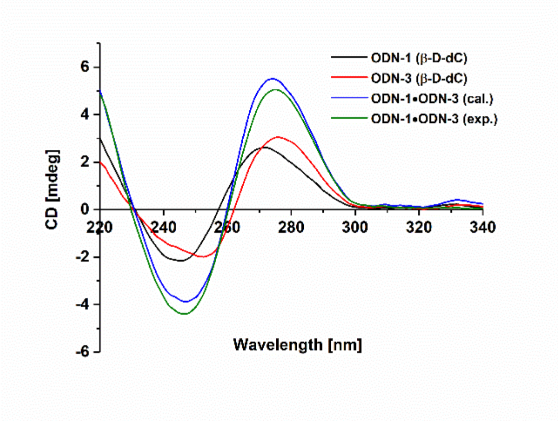

5’-d(TAG GT **C** AAT ACT) (ODN-**1**)
3’-d(ATC CA$^{\alpha}$**C** TTA TGA) (ODN-**7**)

5’-d(TAG GT**C** AAT ACT) (ODN-**1**)
3’-d(ATC CA**C** TTA TGA) (ODN-**3**)

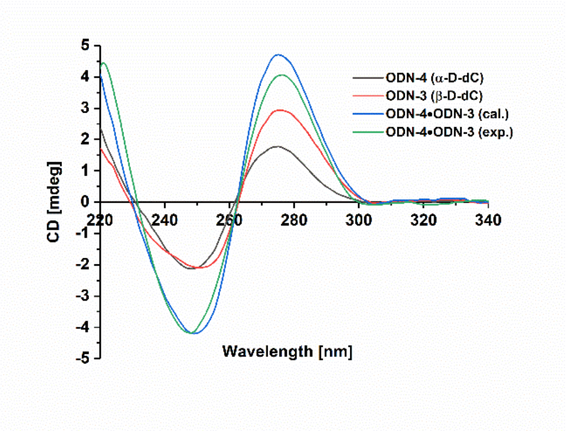

5’-d(TAG GT$^{\alpha}$**C** AAT ACT) (ODN-**4**)
3’-d(ATC CA **C** TTA TGA) (ODN-**3**)

Figure S11. CD spectra of single strands, calculated duplex and experimental duplex. CD measurements were performed in 100 mM NaOAc, 10 mM $Mg(OAc)_2$ buffer (pH 7.4) with 5 µM + 5 µM single-strand concentration. The cell path length of the cuvette was 5 mm.

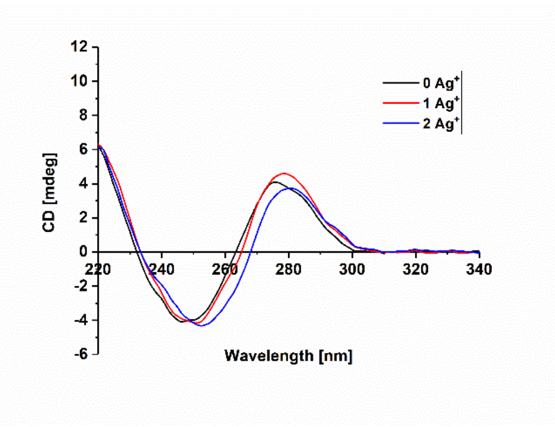

5’-d(TAG GT^{L}**C** AAT ACT) (ODN-**6**)
3’-d(ATC CA$^{\alpha}$**C** TTA TGA) (ODN-**7**)

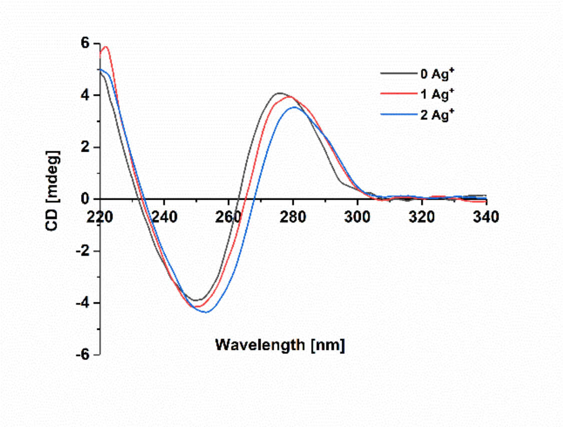

5’-d(TAG GT$^{\alpha}$**C** AAT ACT) (ODN-**4**)
3’-d(ATC CA^{L}**C** TTA TGA) (ODN-**5**)

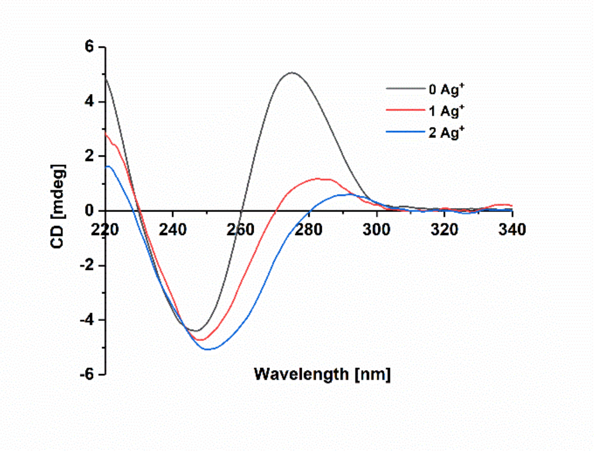

5’-d(TAG GT**C** AAT ACT) (ODN-**1**)
3’-d(ATC CA**C** TTA TGA) (ODN-**3**)

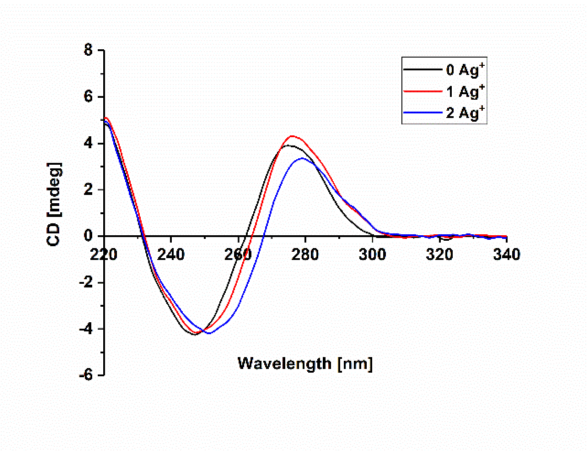

5’-d(TAG GT**C** AAT ACT) (ODN-**1**)
3’-d(ATC CA$^{\alpha}$**C** TTA TGA) (ODN-**7**)

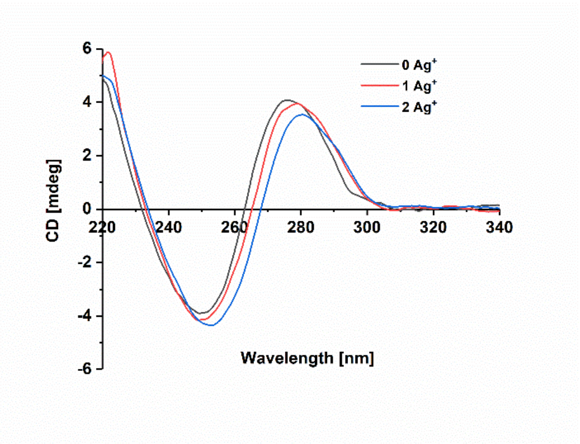

5’-d(TAG GT$^{\alpha}$**C** AAT ACT) (ODN-**4**)
3’-d(ATC CA **C** TTA TGA) (ODN-**3**)

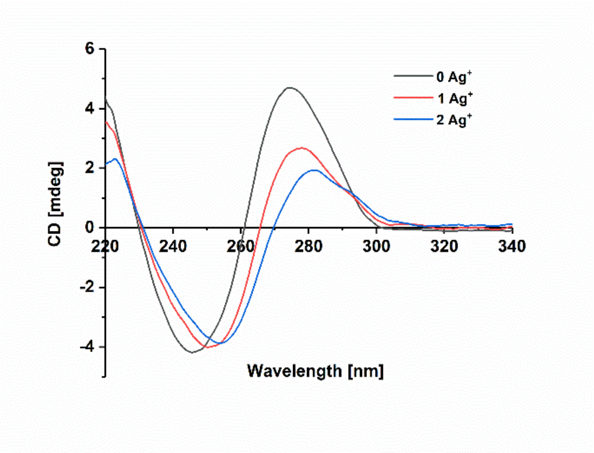

5’-d(TAG GT **C** AAT ACT) (ODN-**1**)
3’-d(ATC CA^{L}**C** TTA TGA) (ODN-**5**)

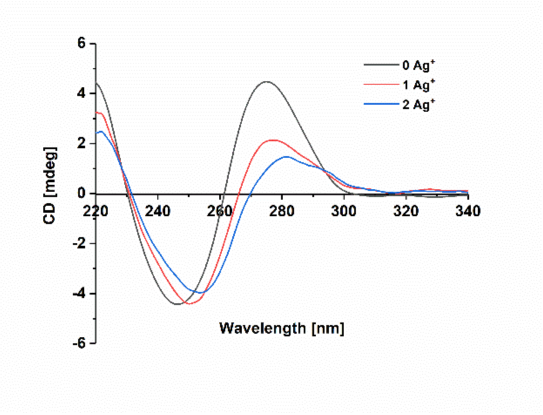

5’-d(TAG GT**^{L}C** AAT ACT) (ODN-**6**)
3’-d(ATC CA **C** TTA TGA) (ODN-**3**)

Figure S12. CD spectra of oligonucleotide duplexes determined in the absence and presence of various concentrations of Ag^+ (0, 1, 2 silver ions/duplex). CD measurements were performed in 100 mM NaOAc, 10 mM $Mg(OAc)_2$ buffer (pH 7.4) with 5 µM + 5 µM single-strand concentration. The cell path length of the cuvette was 5 mm.

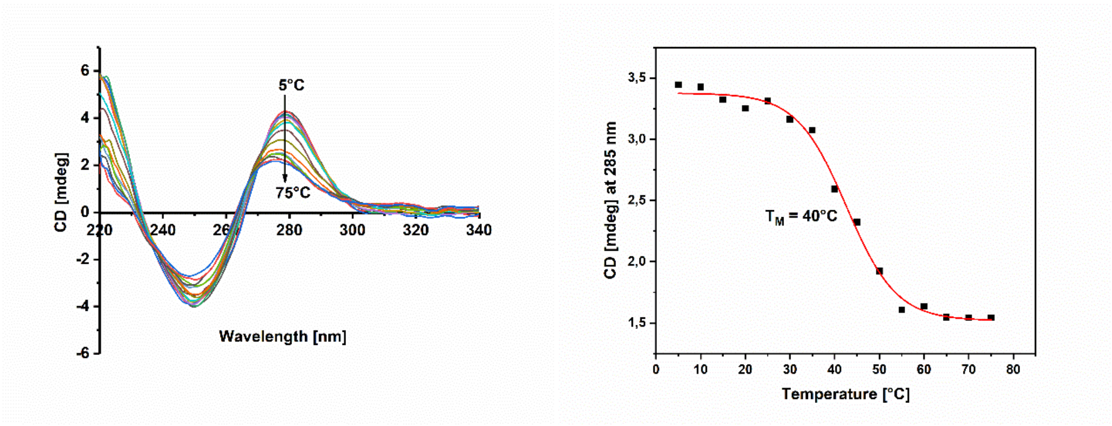

5’-d(TAG GT^{L}**C** AAT ACT) (ODN-**6**)
3’-d(ATC CA$^{\alpha}$**C** TTA TGA) (ODN-**7**)

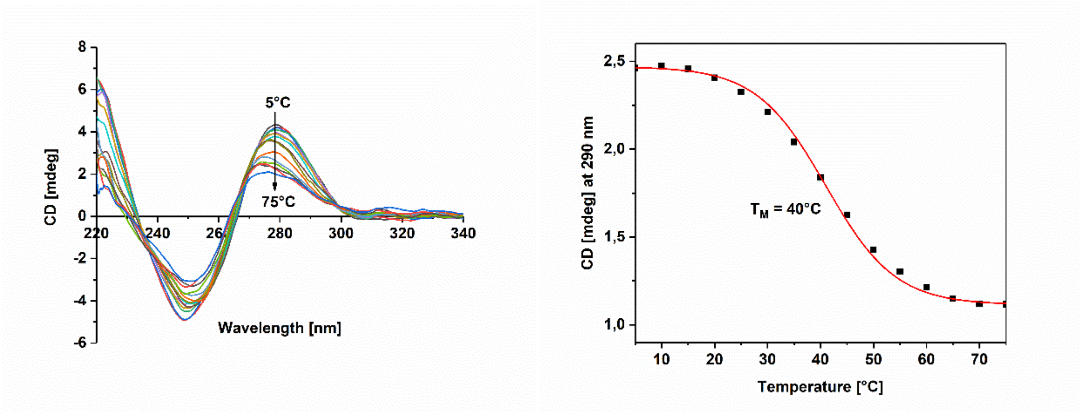

5’-d(TAG GT$^{\alpha}$**C** AAT ACT) (ODN-**4**)
3’-d(ATC CA^{L}**C** TTA TGA) (ODN-**5**)

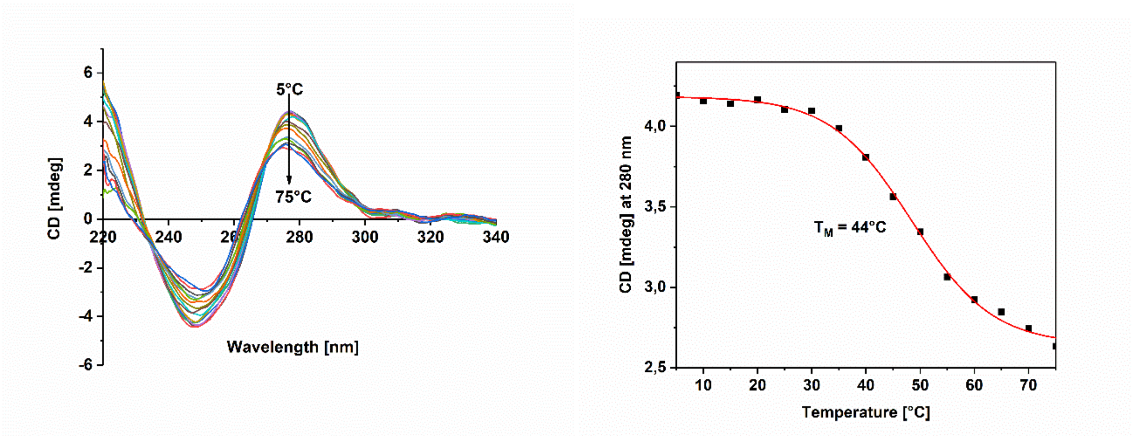

5’-d(TAG GT**C** AAT ACT) (ODN-**1**)
3’-d(ATC CA$^{\alpha}$**C** TTA TGA) (ODN-**7**)

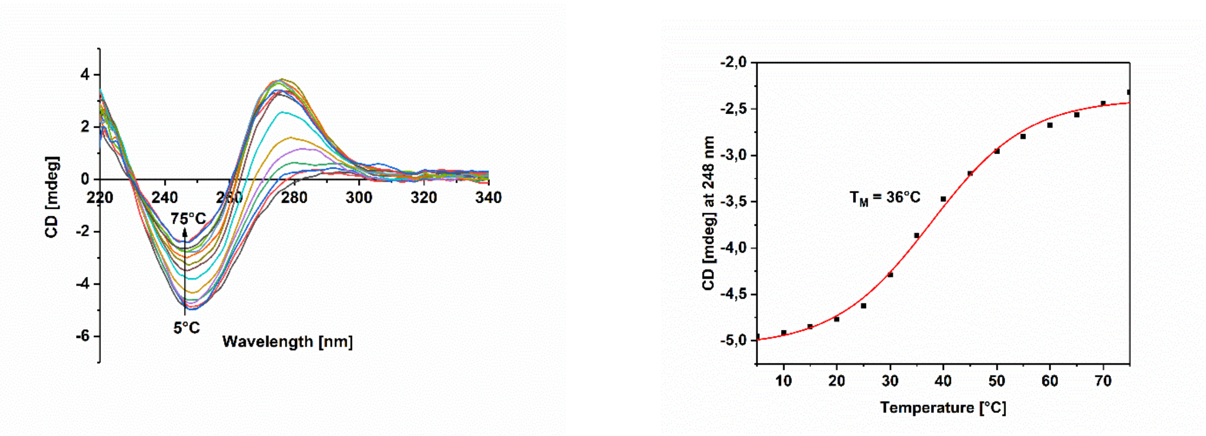

5’-d(TAG GT**C** AAT ACT) (ODN-**1**)
3’-d(ATC CA**C** TTA TGA) (ODN-**3**)

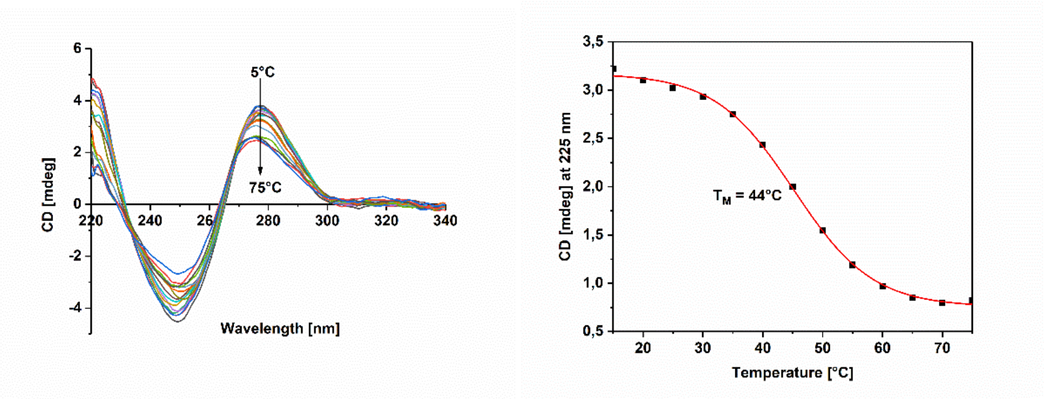

5’-d(TAG GT$^{\alpha}$**C** AAT ACT) (ODN-**4**)
3’-d(ATC CA **C** TTA TGA) (ODN-**3**)

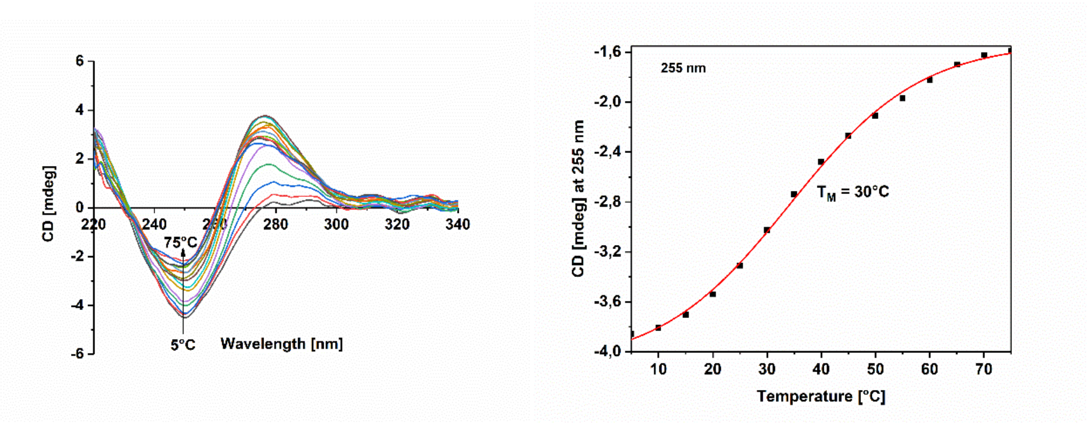

5’-d(TAG GT **C** AAT ACT) (ODN-**1**)
3’-d(ATC CA^{L}**C** TTA TGA) (ODN-**5**)

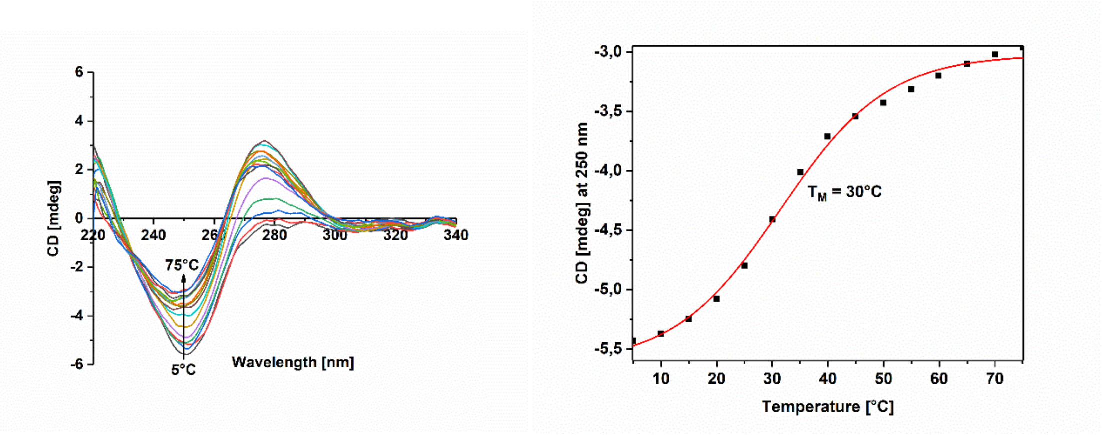

5’-d(TAG GT^{L}**C** AAT ACT) (ODN-**6**)
3’-d(ATC CA **C** TTA TGA) (ODN-**3**)

Figure S13. Left: Temperature-dependent CD spectra. Right: CD melting curves of duplexes in the presence of 1 Ag^+/duplex obtained from temperature-dependent CD spectra. All measurements were performed in 100 mM NaOAc, 10 mM $Mg(OAc)_2$ buffer (pH 7.4) with 5 µM + 5 µM single-strand concentration. The cell path length of the cuvette was 5 mm.
